# Experiments on Galvanic Contractions Excited upon Warm and Principally upon Cold Blooded Animals

**Published:** 1803-03-01

**Authors:** Martin Tupper

**Affiliations:** Exeter College, Oxford


### EXPERIMENTS *on* GALVANIC CONTRACTIONS *excited upon warm and principally upon cold blooded Animals; by* MARTIN TUPPER, *of Exeter College, Oxford.*

[ Continued from our last, pp. 324. ]

EXP. 14. GALVANIC contractions were excited when the armature of the nerves was rubbed with metal, provided a nerve was at the same time touched with it. In these experiments the nerve should be sufficiently long to prevent the contact of the muscle with their armature. When the nerves were placed on glass, or on any other non-exciting substance, and treated in a similar manner, no effects whatever ensued when the limbs had been prepared some time, but the contractions took place when the nerves were replaced on their armature. Contractions were frequently excited when the armature of the nerves was alone rubbed with metal, but such contractions were equally strong when the limbs, nerves, or armatures were touched, or rubbed with the finger, glass, amber, &c. and therefore depended upon simple mechanical irritation.

When the two axillary nerves and the upper limbs were armed, and one of the former was held in the steel forceps and treated in the manner before mentioned, the contractions were very strong in the two limbs, but they were much more powerful in that limb, the nerve of which was at rest upon its armature.

EXP. 15. It has been asserted that muscular contractions were produced when a bar of zinc was dropped upon the armature of the nerves, composed of the same metal. I have repeatedly tried experiments of this nature, and have diversified them in many ways, but never with the success that has been mentioned; I however, at an early period of these experiments, supposed that positive effects had been produced when the sciatic nerves and the lower limbs of a frog had been disposed on their respective armatures, and the armature of the former was rubbed with one extremity of the silver excitatory arc. But it was soon observed, that the plate of glass upon which the apparatus was disposed, and upon which the other extremity of the arc rested was slightly moist on its surface; and this experiment having been repeated when all moisture had been excluded, the effects were negative. I have obtained positive results by dropping a bar of zinc or of silver upon the the armature of the nerves, when one end of either of the two metals was in contact with a circle of water that communicated with the silver armature; I have likewise always been successful when I touched a moist part of the zinc armature with any metal, provided that a circle of water was continued as far as the silver armature; but whenever that was in any manner interrupted, no effects whatever were produced. It appears probable that the mechanical concussion excited in the limbs by the contact of the two metals was sufficient to excite contractions in the muscles, which may have been mistaken for Galvanic effects. I have frequently observed when the nerves and limbs of different animals were disposed upon their respective armatures, that the simple agitation of the table has often produced very violent contractions; and in such instances the same effects took place if the armature of the nerves or of the muscles was struck with any metal.

Exp. 16. The friction of the two extremities of the excitatory arc upon the armatures increases the force of Galvanic contractions very much; especially when the limbs are in a state of weak excitement. Let the nerves and limbs be armed, and let each extremity of the silver arc be very gently made to complete the circle, and no contractions will be excited; but if when the circle is thus completed, either end of the arc be slightly rubbed against the armatures, the results will at once be positive. It cannot be supposed that so slight a friction can produce a degree of heat sufficient to cause this difference in the results; and indeed, the following experiment, in which an arc of water was made use of, in some degree strengthens the doubt. Having placed the armatures upon which the lower limbs and sciatic nerves of a frog were disposed, at the distance of about one-twelfth of an inch from one another, I let fall a drop of water from some height upon them, and very strong contractions were the immediate consequence in the two limbs; but when it was gently dropped, no effects whatever were produced. The simple vibration produced amongst the particles of the metals had no influence over these effects, for when a piece of glass, amber, or wax, was rubbed or struck against the two armatures at the same time, no effect whatever was produced.

Exp. 17. Galvanic contractions are stronger when the wire in the compound excitatory arc is firmly grasped than when it is slightly held in the hand.

Having composed an excitatory arc of a bar of steel, my my body, and a metallic wire, it was invariably found that the greater contact of skin with the metal did not produce this effect, for when the wire was firmly held between the finger and thumb the contractions were much more powerful than when the wire was laid upon the whole length of the naked arm.

Exp. 18. Positive effects are produced when the same metal is made use of in the armatures and excitatory arc. This fact is opposed to the opinion of some philosophers, who have founded a theory upon the necessity of heterogeneity of metals for positive results in Galvanic experiments. I have produced strong contractions in experiments of this kind with quicksilver, zinc, silver, gold, and polished steel.

Exp. 19. Galvanic contractions are continued for a considerable time when the excitatory arc is rubbed without intermission against the armatures. Having disposed the lower limbs and sciatic nerves of a very excitable frog upon their respective armatures, and proceeding in the manner just mentioned, I found that after eleven minutes, the contractions had become considerably weaker; after fourteen they were only partial; after sixteen minutes they had ceased altogether, and could not then be reproduced, unless one end of the silver arc touched any particular muscle, when that alone was excited. When the limbs had rested ten minutes, partial and feeble contractions could be excited; after fourteen minutes they had become a little stronger; after thirty the results had become positive in the two limbs; and after the expiration of forty-two minutes, the muscles had re-accumulated as much excitability as they were capable of at that period of time after death.

Exp. 20. I have produced tolerably strong contractions by arming and exciting the lower extremities of a toad, twenty-six hours after death. The dissected nerves and the toes were so dried up as to be easily crumbled between the fingers, and it appeared that putrefaction had already commenced between the thighs.

Exp. 21. When the sciatic nerves of a frog were armed with gold, and the limbs with zinc, and the circle was completed with iron, silver, or brass, the effects were very powerful; but when the limbs were armed with silver, and the communication was effected as before, the contractions were were comparatively feeble. The nerves and limbs being armed with gold, and the circle being completed with an iron, brass, or silver arc, the effects were tolerably strong. When one point of the iron, brass, or silver arc was gently carried over the limbs till it touched their (gold) armature (the other point resting upon the armature of the nerves) the effects were very strong, and in some respects different from those which are produced when the armatures consist of zinc or silver, or of any other metal except gold; the contractions and relaxations of the muscles in experiments of this kind are extremely quick in succession, they appear rather to flutter than to be affected by the usual strong spasmodic contractions. It has sometimes happened that the results have been negative, when steel, silver, or brass completed the circle upon two gold armatures, whilst at other times they have been positive under apparently similar circumstances. Sometimes no effects were produced when the arc was placed in contact with the (gold) armature of the nerves and with the muscles, whilst they were very strong when the arc was brought into contact with the two armatures; and again, contractions have, in some animals, been excited only when the excitatory arc was connected with the armature of the nerves, and with a piece of muscle placed upon the armature of the limbs. The above facts, which are apparently so contradictory to one another, were made upon animals as nearly as possible of equal degrees of excitability, and with the same pieces of metal.

Exp. 22. *Of the conducting Powers of Charcoal and Coke.*

It is by no means difficult to excite Galvanic contractions in the limbs of animals, when the nerves and muscles are armed with either of the above substances, and the circle is completed from each armature with a metallic excitatory arc. It is necessary however that the charcoal, whether vegetable or mineral, should have been recently and accurately burnt; I tried several pieces of the common charcoal, but succeeded only once in producing contractions when such was employed, but having very carefully charred a piece of deal wood, and proceeding in the usual manner, the effects were very positive, and nearly as strong as I have ever seen them in experiments in which the nerves and muscles were armed with metal.

Some philosophers are of opinion that the non-conducting powers of fresh vegetables, and of common coal, depend upon the hydrogen which forms one of their constituent tuent parts, and which so modifies the carbon as to produce a substance totally insulant. Many experiments however throw some doubts upon this opinion: thus, common charcoal, after having been exposed to heat in close vessels for a considerable time, is almost invariably insulant; and Humboldt has found that the dry carious part of trees was a perfect conductor, although no traces of charcoal could be observed about it. The black lines also which are observed in wood that has been long exposed to water, as well as the charry matter which results from the decomposition of wood by the action of the sulphuric or nitric acids upon it, are perfect insulants in the Galvanic circle.

Exp. 23. *Of Galvanic Contractions excited under Water.*

The sciatic nerves and the lower extremities of a frog, having been armed and immersed in water, the contractions were very strong when the circle was completed. If the upper half of the limbs, together with the nerves and their armature, were held out of the water, the lower half remaining immersed, and the circle was completed from the armature of the nerves to the surface of the water, the effects were likewise positive. When the lower half of the limbs was immersed in water, with which they were connected by seven pieces of brass wire, as many glasses filled also with water, and the circle was completed with a metallic arc from the armature of the nerves to the water in the last glass, the contractions were very powerful in the two limbs. When the arc touched the brass wire, which connected the portions of water together, the effects were more powerful than when the water itself was the substance in contact with the arc.

Exp. 24. *Of the Influence of Galvanism over the involuntary Muscles.*

Some philosophers are of opinion that Galvanism has no effect whatever on the involuntary muscles, and that consequently neither the heart nor the muscular fibres of the intestines can be excited either to more violent or more frequent contractions by using the metallic plates, or even the pile itself. I have repeatedly made experiments on this subject, and have perfectly succeeded in increasing the number and strength of the pulsations of the hearts of cold blooded animals, and more particularly of the auricles, by proceeding in the usual manner with the metallic plates. In several instances the pulsations were increased from between 18 and 22, to between 26 and 30 in a minute; and in one experiment they were increased from 22 to 35. It could not be suspected that mechanical irritation produced these effects, for the points of the metallic arc were not brought into contact with the heart itself but only with the smooth armatures upon which it was laid. In experiments upon this organ it generally happens that its actions may be renewed when they have ceased for some time, and they often continue for one or two minutes after the excitatory arc has been removed from the armatures. I have not attempted to galvanize the hearts of warm blooded animals, and I believe it is doubted by philosophers of the first eminence in this science whether that organ in this class of animals be susceptible of the Galvanic influence; Dr. Fowler however made some experiments, in which I believe he fully succeeded in increasing both the force and frequency of pulsations; and I have frequently excited very strong peristaltic motions in the intestines and stomachs of oxen and of sheep by simply using a silver and zinc plate, and a silver excitatory arc.

Exp. 25. *Of the Sensations of Light excited by disposing Metallic Substances in various Ways.*

It will not be my object to enumerate the effects which are excited in the different organs by the application and connection of metals to various parts of the ramifications of the same nerve, nor the powerful shocks excited by the pile upon different parts of the body; I will very briefly state that the sensation of light may be excited in four ways: First, by applying one piece of metal to the Schneiderian membrane of the nose, the other to either of the eyes. Secondly, or, one to the gum of the upper jaw, the other to the tongue. Thirdly, or, one to the tongue, the other to either of the eyes. And fourthly, by applying one armature to each eye, and producing a communication between them by any metallic substance.

It is difficult to explain in what manner the contact of metals placed as above mentioned produces the sensation of light. There is, I believe, no communication between the internal nasal branch, nor the twigs of the superior maxillary nerve which are distributed to the gums of the upper jaw, nor between the true gustatory, nor gustatory and the optic nerve; nor between the tunica conjunctiva (the only part of the eye to which the armature can be applied) and the same nerve, except it be by the medium of the ciliary plexus which is in part formed from the first branch of the fifth pair of nerves, which branch also supplies plies the tunica conjunctiva. Granting however that nervous communication existed, the phænomena of excited light in the retina could not be explained by it any more than those of excited contractions in the muscles by the armatures and the excitatory arc.

Exp. 26. *Of the conducting and non-conducting Powers of various Substances.*

In the following enumeration, the liquids made use of were contained in small glass tubes having a moveable wire at each extremity, and which could be approximated at pleasure.

My experiments on this subject have led me to conclude with others, that great differences exist in the conducting powers of the various liquids and solids, but that the appreciation of their effects in the arc is extremely difficult, since we can only arrive at a true comparison of them, by observing the phenomena upon animals; these often present so many unforeseen effects, and are so differently affected by the Galvanic influence, although apparently of equal degrees of excitability, that the point cannot be ascertained with precision.

All the metals, and nerves organically united to the body without any contact whatever of metals, are exciters.

The following substances are passive or simply conductors, though some of them may possibly be exciters under extreme degrees of excitability: The living human body; newly burnt charcoal and coke; muscular flesh of all kinds, whether raw, boiled, or dried; water, alkohol, the saturated solutions of the alkalies in water, the ethers, the acids, the solutions of gums in water, of gum resins in proof spirit, and of resins in rectified spirits of wine, liquid soap made either with the fixed or the volatile alkalies, blood, milk, urine, bile, saliva, &c.

The following substances are non-conductors, viz. dried cuticle, common charcoal, mineral coal, dry wood, cork, wax, amber, glass, butter, the essential oils, the fixed oils, sugar, soot, flax, paper, cotton, &c.

*New Burlington Street, February 7, 1803.*

